# Exploring the epidemiological characteristics of Mpox in the Arab Gulf countries

**DOI:** 10.1038/s41598-025-99252-w

**Published:** 2025-05-06

**Authors:** Yehya M. Althobaity, Michael J. Tildesley

**Affiliations:** 1 Department of Mathematics, Taif University, P.O. Box 11099, Taif, Kingdom of Saudi Arabia; 2 The Zeeman Institute for Systems Biology and Infectious Disease Epidemiology Research, School of Life Sciences and Mathematics Institute, University of Warwick, Coventry, CV4 7AL UK

**Keywords:** Ecology, Ecology, Diseases, Health care, Medical research, Risk factors, Mathematics and computing

## Abstract

In May 2022, Mpox outbreaks emerged in regions where the virus is not traditionally prevalent. This study estimates the mean incubation period, serial interval and the extent of presymptomatic transmission in the Arab Gulf Countries (AGC). The effective reproduction number ($R_e$) is also calculated, as well as the degree of heterogeneity ($K$), using the instant-individual heterogeneity transmission model. We analyze data from 41 confirmed cases for which we have complete information, estimating the mean incubation period using gamma, Weibull and lognormal distributions, with respective means of 8.52 (95% CI 7.26–9.98), 8.57 (95% CI 7.28–10.01), and 8.64 (95% CI 7.23–10.26) days. The mean serial interval, based on 31 case pairs, was 7.19 days (95% CI 4.11–12.95), 7.16 days (95% CI 5.80–8.90), and 10.0 days (95% CI 6.30–16.3) for the gamma, Weibull, and lognormal distributions, respectively. The Akaike Information Criterion (AIC) validated the best-fitting models. The serial intervals were shorter than the incubation periods, suggesting that pre-symptomatic transmission occurred in 60% of transmission events. We estimated $R_e$ to be 0.95 (95% highest posterior density [HPD]: 0.93–1.35) and $K$ to be 1.52 (95% HPD: 1.07–5.76), indicating supercritical Mpox transmission in the AGC with limited transmission heterogeneity. Using a Bayesian framework with non-informative priors and a negative binomial distribution, we projected $R_e$ to remain between 0.95 and 1.0 from August 2022 to January 2023, underscoring the need for continued efforts to reduce transmissibility. These findings provide valuable information for public health interventions, emphasizing a multifaceted approach to managing Mpox transmission.

## Introduction

Since May 2022, Mpox (monkeypox) outbreaks have been observed in various countries, especially in Europe, where the virus is not endemic^1^. Essential public health measures to control the spread of the infection include actively identifying cases, tracing contacts, and isolating or quarantining individuals in close contact. The upper bound incubation period for Mpox is estimated to be 21 days, prompting public health authorities to recommend ongoing monitoring and isolation/quarantine of close contacts for at least 21 days after their last known exposure^1,2^. In August 2024, there were a total of 97,786 confirmed cases and 207 deaths in 116 countries^3^. The outbreak manifested predominantly as rapid transmission within the gay community, bisexuals, and other men who have sex with men (MSM). This escalation prompted the WHO to declare a public health emergency of international concern on 23 July 2022.

The incubation period of Mpox is notably influenced by the mode of transmission^4^. As a result, it becomes imperative to elucidate the distribution of the incubation period in the context of recent outbreaks. The outbreaks documented in 2022 and 2023^2,5^ have involved a substantial number of cases with no documented travel histories to regions where Mpox is known to be endemic. Assessing the incubation period in the AGC is particularly important because most of these cases involve travelers, not individuals of the local population. This suggests that the transmission likely began with external sources of infection before becoming locally established. Furthermore, some of these cases involve individuals who self-identify as engaging in same-sex relationships, commonly referred to as men who have sex with men (MSM)^6^. This demographic aspect adds a layer of complexity to understanding the epidemiological dynamics and transmission pathways of Mpox within populations.

Given the ongoing exploration of various aspects of Mpox, particularly its novel mode of transmission, several essential characteristics remain to be fully understood. One such critical aspect is the serial interval, which represents the duration between the onset of symptoms in primary and secondary cases^7^. Estimating the serial interval in the AGC is particularly important and sensitive because many early cases were travelers, not from the local population. Understanding the serial interval is paramount as it not only informs the reproduction number but also guides the intensity of control measures necessary to contain an outbreak, including the potential for pre-symptomatic transmission.

The distributions of the serial interval and incubation period provide valuable insights into pre-symptomatic transmission, which refers to viral spread before clinical symptoms appear. Research indicates that pre-symptomatic transmission plays a significant role in the spread of Mpox^8,9^, highlighting the importance of accurately quantifying its contribution to overall transmission dynamics^8^. Distinct outbreak clusters are particularly informative for understanding how Mpox spreads in populations with no prior exposure to the virus.

The effective reproduction number ($R_e$) provides an average estimate of disease transmissibility but may obscure individual variations in transmission, known as heterogeneity ($K$). High heterogeneity (i.e., $K < 1$) complicates the interpretation of $R_e$ estimates, as localized outbreaks can still occur even when $R_e < 1$, due to the presence of super-spreaders-individuals who transmit the disease to many others. This phenomenon was observed during the 1980–1984 Mpox epidemic in West Africa. There is currently no consensus on the heterogeneity of Mpox transmission for the 2022–2024 outbreaks. It is essential to assess heterogeneity in the AGC for accurate analysis.

This study aims to estimate the incubation period of Mpox by analyzing reported cases from July to August 2022, focusing on key temporal markers such as the dates of symptom onset, exposure start, and exposure end. By examining these details, the study seeks to understand the interval between exposure and symptom onset, which is crucial for understanding disease transmission and informing public health strategies. Additionally, the study investigates the serial interval by analyzing case pairs to assess disease spread, and provides insights into the temporal dynamics of Mpox transmission. Another objective is to estimate the proportion of transmission attributable to pre-symptomatic individuals, using the distributions of the incubation period and serial interval. Furthermore, the study employs an instantaneous transmission model of individual heterogeneity to estimate the effective reproduction number ($R_e$) and the degree of heterogeneity ($K$) for the AGC epidemic. This enables us to simulate monthly infection sizes from August 2022 to January 2023, and compare these simulations with reported data to project future transmission trends. Through detailed analysis and simulation, we seek to deliver actionable insights that will guide public health strategies and policy decisions, ultimately improving the management and control of the ongoing Mpox outbreak.

## Material and methods

### Details of the Mpox datasets

To estimate the incubation period of Mpox, we analyzed data provided by the AGC public health authorities. The dataset included demographic details (e.g., age and gender), exposure dates, and symptom onset dates. Our analysis focused on 41 confirmed cases, all of which had at least one recorded exposure date (either start or end) and complete symptom onset information. These cases originated from Saudi Arabia (15 cases), Bahrain (2 cases), Qatar (5 cases), the UAE (16 cases), and Oman (3 cases). In cases of data conflict or incompleteness, such as when the earliest exposure date was undefined, it was assumed that exposure occurred within 31 days prior to symptom onset^10^. Similarly, in the absence of a date for the latest exposure, exposure was presumed to have occurred before symptom onset. For cases with a history of travel or known contact with an infected source, exposure windows were determined based on the available information.

To estimate the serial interval, we analyzed data from 31 cases with complete information within clusters in Saudi Arabia and the United Arab Emirates, excluding 5 cases due to incomplete data. These cases included both primary (index) and secondary cases, with symptom onset dates available for both, enabling the serial interval to be calculated. Some of these cases were also used in the incubation period estimation, leading to partial overlap in the data for the two analyses, while others were exclusive to one analysis. As the data were collected during the early phase of the outbreak, our analysis focused on direct transmission routes-specifically, transmission directly from primary cases to their secondary cases. This approach excluded potential unobserved intermediate cases and indirect transmission routes, ensuring the analysis captured direct linkages reflected in the data.

Following the approach of^11^, we estimate the proportion of pre-symptomatic transmission by calculating the fraction of instances where the serial interval is shorter than the incubation period. To account for their potential correlation, we incorporate covariance into our method. The mean difference between these variables is the difference in their means, providing an estimate of the average transmission time occurring before symptom onset. The distribution of this difference depends on their covariance, which we approximate using paired serial interval and incubation period estimates from case data. Sampling from their distributions while preserving the observed correlation allows us to analyze these differences and estimate the fraction of pre-symptomatic transmission.

To apply the instantaneous transmission model of individual heterogeneity to estimate the effective reproduction number ($R_e$) and transmission heterogeneity ($K$) in the Mpox epidemic, $K$ quantifies how transmission is distributed among individuals. A small $K$ (e.g., $K < 1$) indicates high heterogeneity, with a few individuals driving most secondary infections, while a large $K$ (e.g., $K > 1$) suggests more uniform transmission. Lower $K$ values highlight “super-spreading” events. This analysis used daily incidence data for confirmed cases of Mpox in Saudi Arabia, Bahrain, Qatar, UAE, and Oman, with diagnosis dates as reporting dates. Days with no reported cases were accounted for without affecting the estimation of transmission dynamics.

To project Mpox transmission dynamics in the AGC from August 2022 to January 2023, we simulated infections using constant values for the effective reproduction number ($R_e$) and the dispersion parameter $(K)$. Based on the observed incidence curve (July 28–August 17) and an individual-instant heterogeneity model, daily infections were simulated and aggregated into monthly totals. We tested $R_e$ values of 0.8, 0.85, 1.0 and 1.05, together with $K$ values of 0.5, 1.0, 1.5, and 2.5. For each combination of parameters, 50,000 simulations were performed to estimate the median case numbers, which were then compared with the official counts to project the potential transmission dynamics.

## Statistical analysis

### Incubation period

We employed contact tracing information and exposure interval censoring to analyze the incubation period of Mpox, utilizing survival analysis techniques to handle interval-censored data. Specifically, each individual’s exposure window was defined based on contact tracing data, providing the earliest possible exposure time $\varvec{E_L}$ and the latest possible exposure time $\varvec{E_R}$. Using the exact symptom onset date $\varvec{S}$, we defined the incubation period $\varvec{T}$ as a non-negative, continuous random variable with probability density function $f_{\varvec{\theta }}(t)$, dependent on a parameter vector $\varvec{\theta }$.

Given the exact symptom onset time $\varvec{S}$ and exposure window $\varvec{E_L}, \varvec{E_R}$, our data are single interval-censored, with the interval $(\varvec{T_L}, \varvec{T_R}) = (\varvec{S} - \varvec{E_R}, \varvec{S} - \varvec{E_L})$. This interval provides a range within which the incubation period $\varvec{T}$ falls, although the precise value remains unknown. For single interval-censored data, likelihood can be expressed as:

1 $$\begin{aligned} L(\varvec{\theta }; \varvec{T_L}, \varvec{T_R}) = \int _{\varvec{T_L}}^{\varvec{T_R}} f_{\varvec{\theta }}(t) \, dt = F_{\varvec{\theta }}(\varvec{T_R}) - F_{\varvec{\theta }}(\varvec{T_L}) \end{aligned}$$

Here, $F_{\varvec{\theta }}(t)$ represents the cumulative distribution function (CDF) corresponding to the probability density function $f_{\varvec{\theta }}(t)$, parameterized by $\varvec{\theta }$.

We used the *survival* package to compare the Kaplan-Meier nonparametric estimate with three parametric models commonly used in survival analysis-gamma, Weibull, and lognormal-to fit the incubation period distribution. These models were selected due to their suitability for non-negative variables and frequent application in interval-censored data analysis. For each individual $i = 1, 2, \ldots , n$, we represented the incubation period using interval-censored data $[\varvec{T_{L_i}}, \varvec{T_{R_i}}]$, where $\varvec{T_{L_i}} < \varvec{T_{R_i}}$. The likelihood function for each parametric model is:

1 $$\begin{aligned} L (\varvec{\theta }; \cdot ) = \prod _{\varvec{T_{L_i}} < \varvec{T_{R_i}}} \left( F_{\varvec{\theta }}(\varvec{T_{R_i}}) - F_{\varvec{\theta }}(\varvec{T_{L_i}}) \right) \end{aligned}$$

To estimate the 95% confidence intervals (CIs) for the incubation period, we employed a parametric bootstrap approach. Specifically, we resampled the data 10,000 times based on the fitted parametric model, recalculating the incubation period distribution for each iteration. For each resample, the incubation period was computed as the interval within which 2.5% and 97.5% of the resampled estimates fell, thereby defining the 95% CIs. This method ensures that the uncertainty inherent in the parameter estimates is accurately reflected in the reported confidence intervals.

To select the model that best fits the incubation period data, we used the Akaike Information Criterion (AIC), which considers both model fit and complexity. This approach provides a robust selection criterion, allowing us to identify the most appropriate parametric model for our interval-censored data.

### Serial interval

Estimating the serial interval requires identifying infector–infectee pairs, often through contact tracing, which is resource-intensive and challenging during widespread Mpox transmission^12^. Most studies focus on well-defined networks, such as households or sexual contacts, which may not represent broader transmission dynamics. We assume that symptom onset times are available for linked cases; however, missing cases, prolonged viral shedding, and indirect transmission introduce uncertainty. Thus, we distinguish between the “true serial interval”—the time between symptom onset in a directly linked pair—and the “observed serial interval,” which may be influenced by indirect or coprimary transmission.

If we identify case $i$ and case $j$ as connected, with $i$ showing symptoms before $j$ (denoted as $i \rightarrow j$), and if $i$ directly transmitted the disease to $j$, as is the scenario in our dataset, then the difference between the symptom onset times of the two cases can be modeled using Gamma, Lognormal, and Weibull distributions with the following densities:

- Gamma distribution with density:
  $$f(t;\alpha ,\beta ) = \frac{\beta ^\alpha }{\Gamma (\alpha )} t^{\alpha -1} e^{-\beta t}$$
  where $$t > 0$$, $$\alpha > 0$$, and $$\beta > 0$$.
- Lognormal distribution with density:
  $$f(t;\mu ,\sigma ) = \frac{1}{t\sigma \sqrt{2\pi }} e^{-\frac{(\ln (t) - \mu )^2}{2\sigma ^2}}$$
  where $t > 0$, and $\mu$ and $\sigma$ are the mean and standard deviation of the natural logarithm of *t*, respectively.
- Weibull distribution with density:
  $$f(t;\lambda ,k) = \frac{k}{\lambda } \left( \frac{t}{\lambda }\right) ^{k-1} e^{-(\frac{t}{\lambda })^k}$$
  where $$t > 0$$, $$\lambda > 0$$, and $$k > 0$$.

To estimate the 95% confidence intervals (CIs) for the parameters of the fitted distribution, we utilized the bootdist function from the fitdistplus package. This function implements a parametric bootstrap approach, resampling the data based on the fitted parametric model and re-estimating the parameters for each resample. The 95% CIs were derived as the range between the 2.5th and 97.5th percentiles of the bootstrapped parameter values. This method accounts for the joint uncertainty distribution of the parameters, ensuring robust and accurate interval estimation.

To identify the best fit for the serial interval, we used the Akaike Information Criterion (AIC), which helps evaluate the trade-off between model fit and complexity.

### Estimation of pre-symptomatic transmission

Following the methodology outlined in^11^, we first sampled incubation period parameters using data fits from the main text, incorporating variance between shape and scale parameters of the gamma distribution. This yielded 100 incubation period (shape, scale) pairs. We generated 100 gamma-distributed serial interval (shape, scale) pairs and, for each, sampled 500 joint incubation periods and serial intervals with a correlation of approximately 0.38. This produced 50,000 joint samples. The proportion of instances where the serial interval minus the incubation period was negative provided an estimate of the fraction of pre-symptomatic transmission, accounting for covariation.

### Estimation of the effective reproductive number $R_e$

This analysis utilizes the individual-instant heterogeneity model as described in^13^ to analyze incidence data, facilitating the simultaneous estimation of $R_e$ and $K$. Let $I_t$ denote the daily incidence data at time $t$, and $\{I_1, I_2, \ldots , I_{t-1}\}$ represent the epidemic curve up to time $t$. This model captures the emergence of new infections through a branching process with a negative binomial offspring distribution:

$$I_{t} | \bar{I}_{1}^{t-1} \sim \text {NegB} \left(K \Lambda _{t}, \frac{K}{K + R_e} \right).$$

Thus, the probability mass function is given by:

$$P(I_t) = \left( {\begin{array}{c}I_t - 1 + K \Lambda _t\\ K \Lambda _t - 1\end{array}}\right) \left( \frac{R_e}{K + R_e} \right) ^{I_t} \left( \frac{K}{K + R_e} \right) ^{K \Lambda _t}.$$

Here, $\Lambda _t = \sum _{s=1}^{t-1} I_s w_{t-s}$ represents the total infectiousness at time $t$. The weight $w_{t-s}$, also known as the infectiousness profile, defines the impact of each past case on new infections and can be approximated using the serial interval distribution. The individual-instant heterogeneity model extends the classical branching process model with a Poisson offspring distribution by incorporating individual-level transmission heterogeneity, represented by $K$.

We employed a Bayesian framework with non-informative priors-uniform distributions for $R_e$ and $K$-to estimate posterior distributions based on Mpox incidence data from the AGC. For each posterior distribution, we identified the maximum a posteriori estimate and calculated the 95% highest posterior density (HPD) interval. The HPD interval was determined as the narrowest interval containing 95% of the posterior distribution’s density, ensuring that all points within the interval had higher posterior density than those outside it.

## Software and analysis tools

Our data analysis and model fitting were conducted using R (version 4.1.0) and several R packages, including survival for time-to-event data, icenReg for interval-censored data, and fitdistrplus for parametric model fitting, along with PyMC in Python for Bayesian inference. This toolkit enabled effective modeling of Mpox transmission dynamics. We employed the survival package (version 3.5-8) for survival analysis, including Kaplan-Meier and parametric survival models, and the icenReg package (version 2.0.16) to estimate parametric distributions for the incubation period. Additionally, fitdistrplus (version 4.1.2) was used to estimate the serial interval and calculate the Akaike Information Criterion (AIC) to assess model fit. We used lcmix to generate multivariate gamma distributions, providing a means to simulate correlated incubation period and serial interval distributions. These tools provided a robust framework for analyzing both the incubation period and serial interval with nonparametric and parametric methods.

## Results

### Incubation period

Mpox incubation periods were estimated by directly measuring the duration between exposure to the virus and the onset of symptoms. This crucial metric helps us understand the dynamics of the disease and its transmission dynamics. Given the variability inherent in incubation periods, particularly in the context of Mpox where factors like individual immune responses and viral strain differences can influence the onset of symptoms, accurate estimation becomes paramount.

**Fig. 1:**
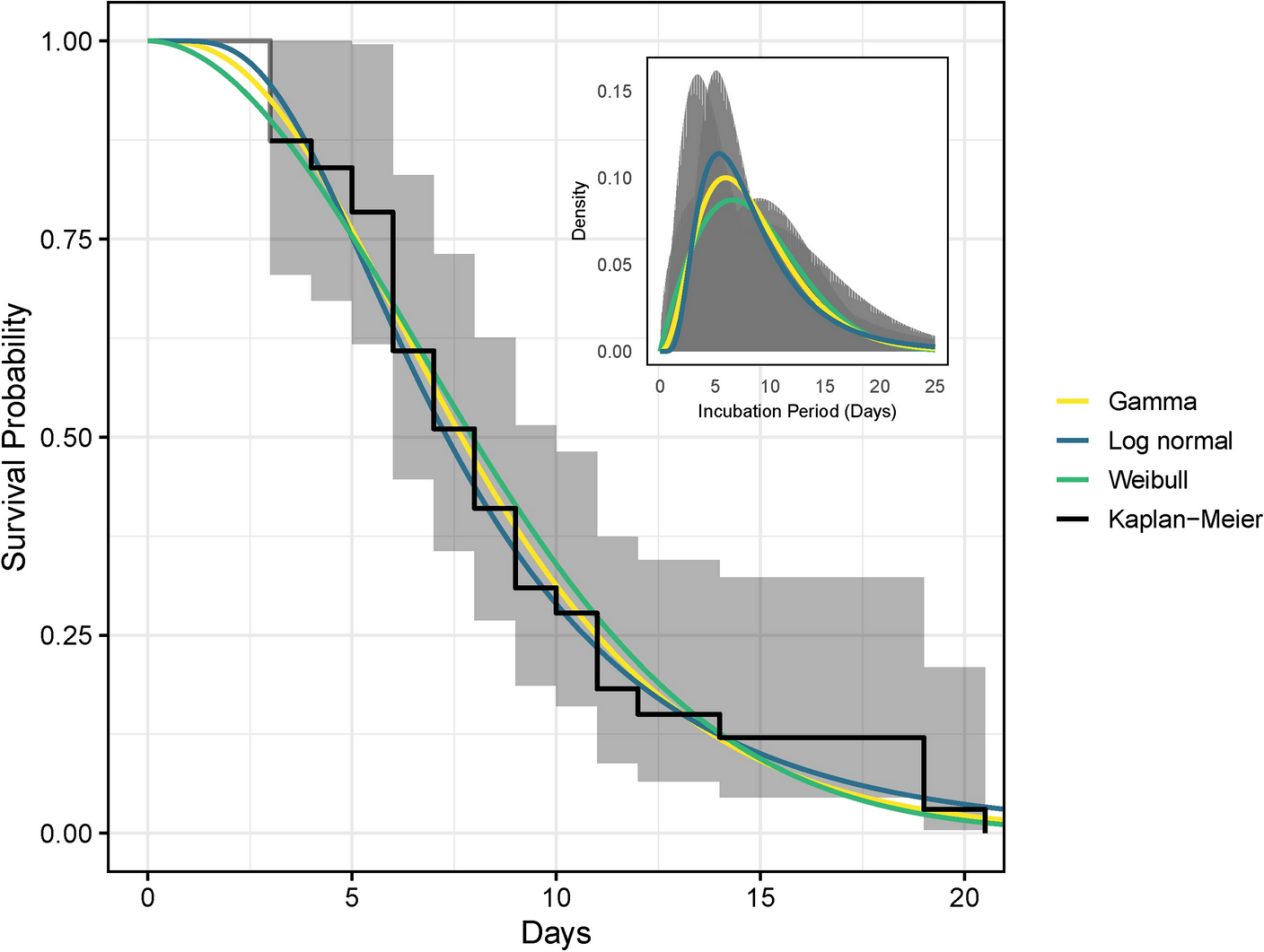
Evaluating the distribution of Mpox infection incubation times involves comparing nonparametric and parametric estimations. This includes contrasting the nonparametric Kaplan-Meier estimate of the incubation period distribution, with its 95% confidence interval (CI) (represented by the gray shaded area), against the parametric lognormal, Weibull, and gamma distributions. The subplot in the top right corner illustrates the density estimation of Mpox incubation periods using gamma, Weibull, and log-normal distributions.

The mean incubation durations were estimated as 8.57 days (95% CI 7.28–10.01) for the Weibull distribution, 8.52 days (95% CI 7.26–9.98) for the gamma distribution, and 8.64 days (95% CI 7.23–10.26) for the lognormal distribution. The AIC revealed that the gamma distribution provided the best fit for the data (AIC = 208.8504), striking the optimal balance between accuracy and simplicity in modeling Mpox incubation periods, as detailed in Table 1. These estimates shed light on the average time for symptoms to manifest after exposure and include associated uncertainty through confidence intervals, illustrated in Fig. 1. This uncertainty must be acknowledged in order to make informed decisions regarding public health interventions and outbreak management.

**Table 1:** Estimates of the incubation period utilizing gamma, Weibull, and lognormal distributions.

Gamma	Mean	Shape	Rate	SD	AIC
	8.52 (7.26–9.98)	3.39 (2.22–4.27)	2.49 (1.59–2.93)	4.58 (2.36–6.05)	208.8504

Weibull	Mean	Shape	Scale	SD	AIC
	8.57 (7.28–10.01)	1.93 (1.53–2.43)	9.61 (8.18–11.28)	4.60 (3.18–6.77)	211.4942

Lognormal	Mean	Mean-log	Sd-log	SD	AIC
	8.64 (7.23–10.26)	1.99 (1.82–2.22)	0.56 (0.44–0.70)	5.19 (3.14–9.35)	209.7080

Confidence intervals for the shape and scale parameters (logarithmic mean and standard deviation for lognormal) are presented in brackets, representing a 95% confidence level. To identify the most suitable distribution, we utilized the Akaike Information Criterion (AIC). The distribution with the lowest AIC value was selected as the best-fitting model among the candidates.

We compare nonparametric and parametric estimations of the distribution of the Mpox infection incubation period through visual methods. Specifically, we contrast the Kaplan-Meier estimate (nonparametric) with parametric models, including the lognormal, Weibull, and gamma distributions. This analysis is based on stratified data that include all cases with symptom onset. Figure 1 displays this comparison, with an inset in the top right corner showing the density distributions for the gamma, Weibull, and lognormal models.

### Serial interval

The infectious period for Mpox is poorly characterized. Serial intervals (time between symptom onset in primary and secondary cases) are often used as a proxy, with studies suggesting over 90% of transmission occurs within two weeks of symptom onset^7^. However, biases from self-reporting and differences between serial intervals and the infectious period complicate these estimates. Recent findings also reveal significant heterogeneity, with viral shedding lasting 23 to 50 days in some individuals^14^. Fixed-duration isolation may therefore risk prematurely releasing long-term infectious individuals or unnecessarily extending isolation for others^14^.

We directly estimate the serial interval distributions by analyzing pairs consisting of a primary case and its corresponding secondary case to determine the time interval between symptom onset in the primary case and in the associated secondary case. In this analysis, we exclude secondary cases with incomplete symptom onset data. This ensures that only those transmission case pairs with reliable symptom onset information are considered. By focusing on these pairs, we aim to provide a more accurate understanding of the temporal dynamics of Mpox transmission. Our analysis includes 36 transmission pairs, and we excluded 5 secondary cases due to incomplete symptom onset data. While this approach provides valuable insights into the disease’s epidemiology, it is important to note that our estimates reflect the observed variability within the available sample, rather than the full range of potential variability in the broader population. This understanding helps inform the development of effective public health interventions.

**Fig. 2:**
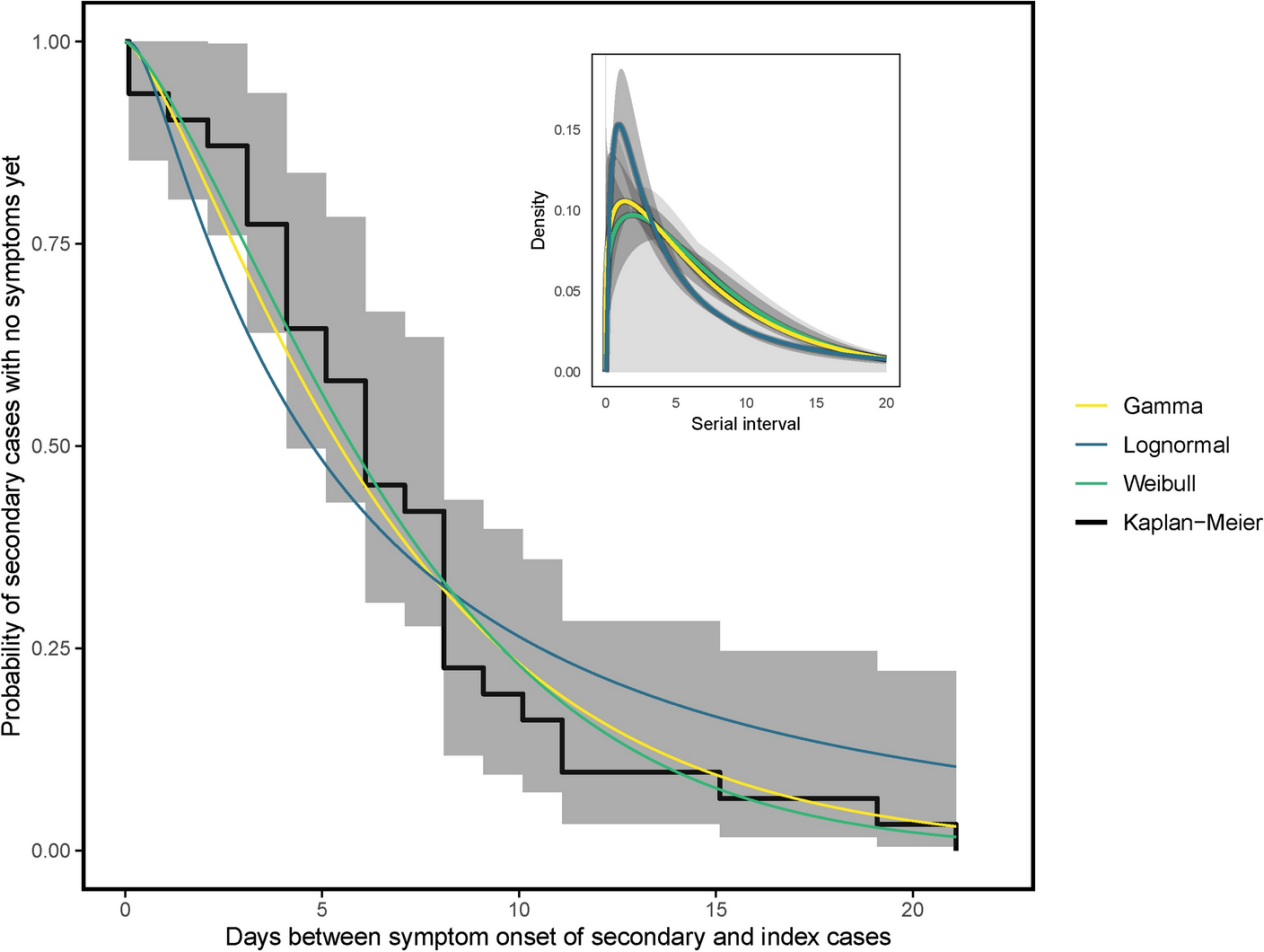
Evaluating the distribution of Mpox infection serial intervals involves comparing nonparametric and parametric estimations. This includes contrasting the nonparametric Kaplan-Meier estimate, with its 95% confidence interval (CI) (represented by the gray shaded area), with the parametric lognormal, Weibull, and gamma distributions. The subplot in the top right corner illustrates the density estimation of Mpox serial intervals using gamma, Weibull, and lognormal distributions.

Within the scope of our investigation, we identified the largest clusters of Mpox cases in the UAE and Saudi Arabia, comprising 16 and 15 cases, respectively. Notably, some of these cases were primarily among visitors and individuals from other countries, indicating that the virus had possiby been imported into these regions. Our detailed analysis revealed variability in the mean serial interval for Mpox across different statistical distributions. Specifically, the mean serial interval was estimated as 7.19 days (95% CI 4.11–12.95) for the gamma distribution, 7.16 days (95% CI 5.80–8.90) for the Weibull distribution, and 10.0 days (95% CI 6.30–16.3) for the lognormal distribution. Assessment of the goodness of fit using the AIC showed that the Weibull distribution provided the best fit to the data, evidenced by the lowest AIC value (601.3836). These results are summarized in Table 2, while Fig. 2 visually illustrates the estimated serial intervals for Mpox using the lognormal, gamma, and Weibull distributions.

**Table 2:** We estimated the serial intervals employing gamma, Weibull, and lognormal distributions.

Gamma	Mean	Shape	Rate	SD	AIC
	7.19 (4.11–12.95)	1.23 (0.96–1.60)	0.17 (0.12–0.23)	6.51 (4.26–9.82)	604.1365

Weibull	Mean	Shape	Scale	SD	AIC
	7.16 (5.80–8.90)	1.22 (1.05–1.41)	7.69 (6.39–8.94)	5.89 (5.76–5.92)	601.3836

Lognormal	Mean	Mean-log	Sd-log	SD	AIC
	10.09 (6.3–16.38)	1.52 (1.26–1.78)	1.26 (1.08–1.44)	19.9 (9.39–44.0)	645.7677

The 95% confidence intervals for the shape and scale parameters (log mean and standard deviation for lognormal) are provided within brackets. To identify the most suitable distribution, we utilized the Akaike Information Criterion (AIC). The distribution with the lowest AIC value was selected as the best-fitting model among the candidates.

### Pre-symptomatic transmission

**Fig. 3:**
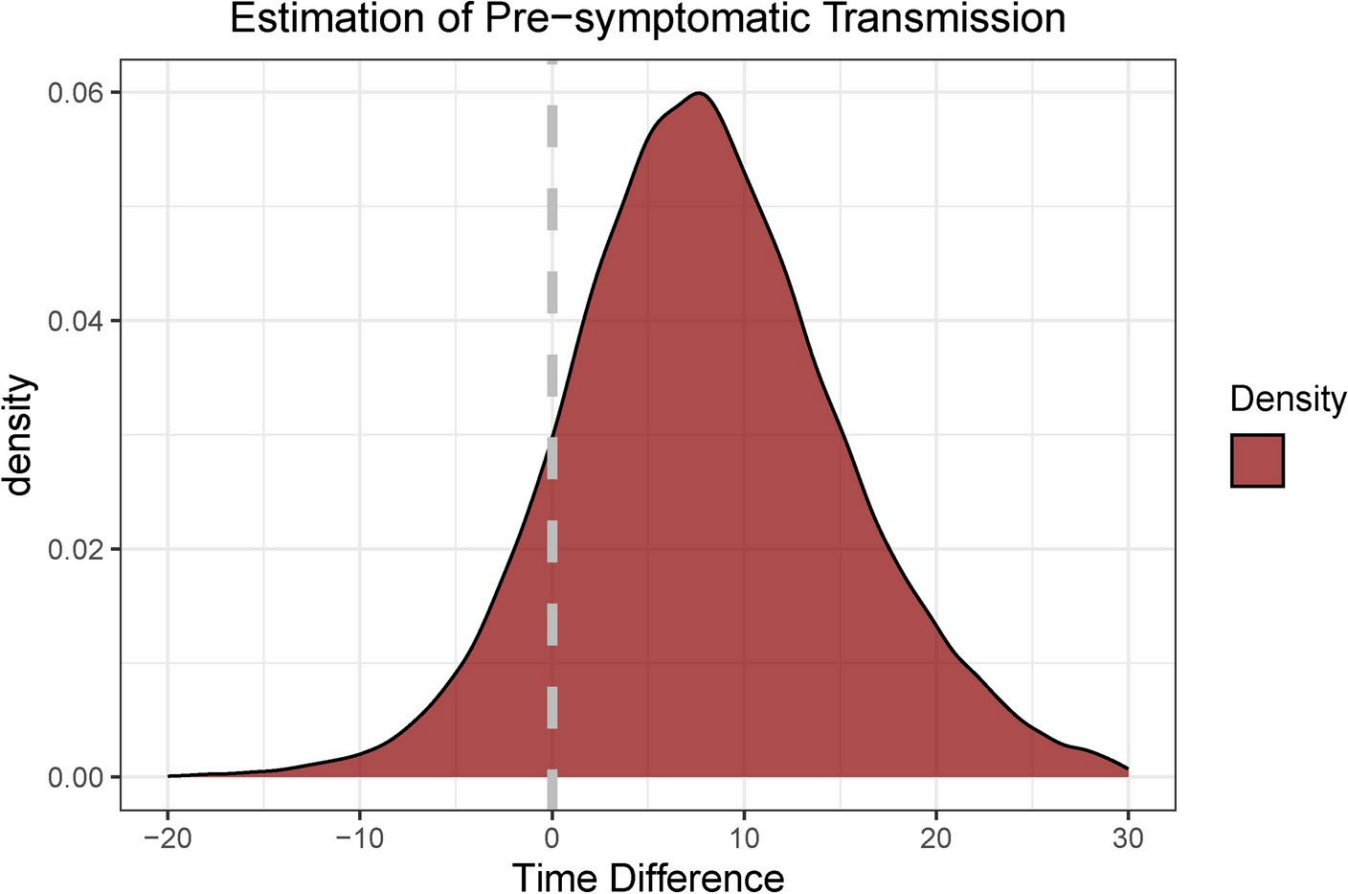
Estimation of pre-symptomatic infection in the AGC for Mpox, based on the difference between serial interval and incubation period samples, accounting for covariation and correlation. The grey vertical line marks the point where the time difference equals zero, with samples below this line indicating pre-symptomatic transmission.

We estimated the incubation periods and serial intervals, observing that the serial intervals were consistently shorter than the incubation periods. This finding suggests pre-symptomatic transmission. Through joint sampling of these intervals, we determined that approximately 61% of transmissions occur prior to symptom onset, based on direct estimates (refer to Fig. 3). Analysis of Mpox data from AGC revealed a covariance of 2.67 and a correlation coefficient of 0.38, highlighting a moderate positive association between these variables.

The relatively high estimate of pre-symptomatic transmission in the AGC may be influenced by the long apparent incubation periods, which could reflect pre-symptomatic transmission during quarantine or assumptions made during the creation of the original dataset. Under reasonable assumptions, our findings suggest that transmission occurs prior to symptom onset in around 61% of cases, with infection on average occurring 1.14 days before the infector’s symptoms appear. Overall, serial intervals are shorter than incubation periods (see Tables 1 and 2), and the fact that both were estimated from the same populations further strengthens these results. Additionally, the differences between incubation periods and serial intervals suggest that a substantial portion of transmission instances happen before symptoms emerge.

### Estimation of the effective reproductive number $R_e$

**Fig. 4:**
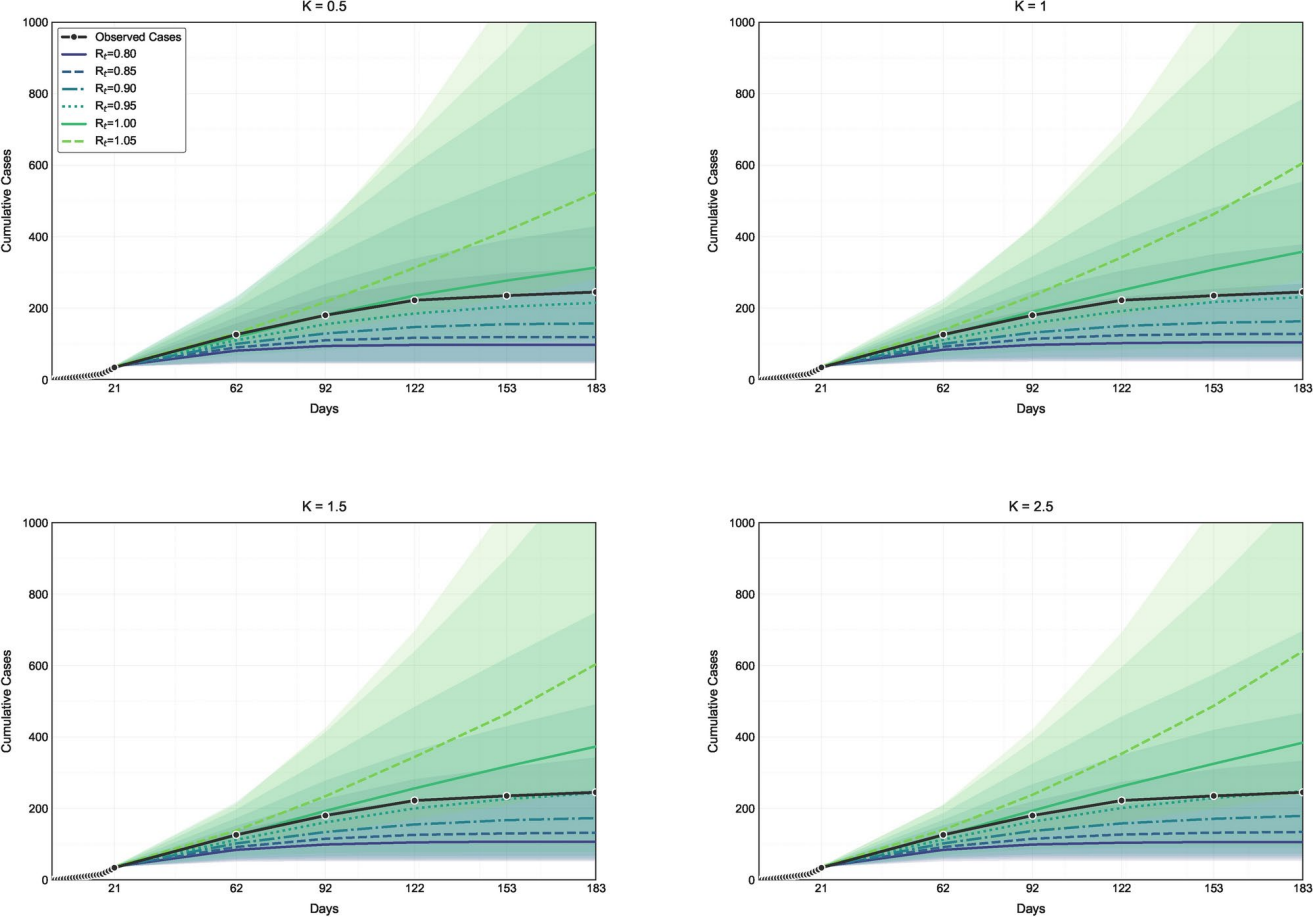
Comparing the cumulative case data with the model projections for the 2022 Mpox epidemic in the AGC provides notable insights. The black lines represent the cumulative case data from June 12, 2022, to January 30, 2023. These data are compared with projections from the individual-instant heterogeneity model, which includes scenarios of homogeneous transmission (panels B–D with $K = 1$, $K = 1.5$, and $K = 2.5$) and heterogeneous transmission (panel A with $K = 0.5$). The colored lines illustrate the median values from the simulated data, reflecting various effective reproduction numbers ($R_e$). The shaded areas around the colored lines represent the 95% uncertainty intervals, providing a visual representation of the variability and confidence in the model projections. The simulations were conducted using a Weibull-distributed serial interval with a mean of 7.16 days and a standard deviation of 5.89 days, and were iterated 50,000 times.

Turning to the specific dynamics of the Mpox epidemic in the AGC, which began on July 28, 2022, we estimated $R_e$ to be 0.95 [95% HPD: 0.93–1.35], indicating that the effective reproduction number was below the critical threshold of 1 (Fig. 4). Moreover, we calculated $K$ to be 1.52 [95% HPD: 1.07–5.76], suggesting that transmission heterogeneity was not significant. Given the ongoing spread of Mpox in the AGC, we employed simulation models to project the future trajectory of transmission. These simulations, which assumed a constant serial interval, showed that the confirmed cases reported between July and August closely aligned with the simulated data, particularly for $R_e$ values of 0.95 and 1.0 (Fig. 4). This indicates that the average $R_e$ during this period likely fluctuated between 0.95 and 1.0, highlighting the evolving dynamics of the epidemic.

To evaluate model robustness and account for uncertainties, we performed a sensitivity analysis using the same methodology as in the main analysis but assumed a gamma distribution for the serial interval. Following the recommendations of^13^, we used a mean of 7.19 days and a standard deviation of 6.51 days. Additionally, we assessed the convergence of the posterior distributions to verify the stability and reliability of the parameter estimates. Further details on the methodology and results can be found in the supporting information.

## Discussion and conclusion

This research analyzed Mpox transmission clusters to estimate key parameters such as the incubation period, serial interval, pre-symptomatic transmission, and effective reproduction number ($R_e$), which are crucial for guiding public health interventions and models of transmission dynamics. These models are vital tools for both local and global responses to Mpox.

Although relatively few studies have examined Mpox incubation periods, recent outbreaks in Europe and the USA have provided key estimates: 8.5 days in the Netherlands, 7.6 days in the UK, and 5.6 days in the USA. Our analysis using Gamma, Log-normal, and Weibull distributions produced estimates ranging from 8.52 to 8.64 days-consistent with the diverse exposure routes observed among men who have sex with men (MSM) presenting with genital lesions. Differences across studies may reflect variation in transmission modes; for instance, the shorter incubation period reported in the USA (5.6 days) may be attributable to a higher proportion of direct intimate contact exposures compared to European cohorts. While our dataset did not allow for formal stratification by exposure type due to inconsistent exposure characterization, these between-study comparisons suggest that the incubation period may vary by transmission route, with more direct exposures potentially leading to shorter incubation times. This hypothesis is consistent with known viral transmission dynamics and underscores the need for future studies with more systematically collected exposure data.

Accurately estimating the serial interval requires identifying multiple infector-infectee pairs, which is typically achieved through detailed contact tracing. However, this process can be resource-heavy and difficult in areas with widespread Mpox transmission. Most studies, therefore, focus on more contained transmission networks, such as households or sexual contacts, which may not fully capture the broader transmission patterns^12^. In our study, we assume that symptom onset times for linked cases are available, although factors like incomplete data, prolonged viral shedding, and indirect transmission introduce some uncertainty. As a result, we differentiate between the true serial interval-the time between symptom onset in directly linked cases-and the observed serial interval, which refers to the time difference between symptom onsets in epidemiologically linked cases. For example, if case *i* develops symptoms before case *j*, we assume case *i* is the infector; however, this assumption may not always be correct due to indirect or coprimary transmission^12^. The estimated serial intervals for Mpox were 7.19 days (gamma), 7.16 days (Weibull), and 10.0 days (lognormal), which are consistent with previous studies but show variation based on demographics and social interactions^15,16^. The shorter serial intervals, particularly among men who have sex with men (MSM), may be due to frequent close contact and better control measures. Additionally, since the serial interval is shorter than the incubation period, it suggests a significant amount of transmission occurs before symptom onset, with at least 51% of cases transmitted pre-symptomatically. This highlights the challenges of controlling Mpox through isolation alone, suggesting the need for broader intervention strategies.

The AGC outbreak was primarily driven by external transmission, and we estimated $R_e < 1$, indicating limited internal spread. However, further infections were likely after January 30, 2023, as $R_e$ approached 1. Our analysis revealed a homogeneous transmission pattern, with 80% of secondary infections originating from 50% of primary cases, deviating from the typical 20/80 rule.

Our study has several important limitations that should be considered when interpreting the results. First, regarding our estimation of serial intervals and incubation periods, we did not employ double-interval censoring methods due to multiple practical and data-related challenges. The highly variable presentation of Mpox symptoms, ranging from delayed rash appearance to nonspecific early symptoms like fever, made precise determination of exposure windows and symptom onset dates particularly difficult. These uncertainties were compounded by incomplete data availability, especially for travel-related cases, and inconsistencies across different national reporting systems. Furthermore, within the complex sexual networks of affected MSM populations, identifying single definitive exposure events was often impossible, as most exposure windows had to be inferred epidemiologically rather than observed directly.

An additional key limitation stems from our methodological assumption that the case with earlier symptom onset was necessarily the infector. While this approach helped reduce misclassification risks associated with unreliable reporting dates, it inherently excluded the possibility of presymptomatic transmission by disregarding negative serial intervals. Although we acknowledge elsewhere (Subsection 6.3) that presymptomatic transmission does occur in Mpox, our dataset lacked the necessary temporal precision in exposure histories or viral load measurements to more accurately determine transmission directionality. This likely introduced an upward bias in our serial interval estimates.

Regarding transmission dynamics in the MSM population during the Mpox outbreak, we recognize the limitations of estimating a time-independent dispersion parameter ($k$) in the context of highly heterogeneous sexual contact networks. These networks exhibit dynamic transmission patterns, where $R_e$ declines and $K$ increases as the susceptible population depletes over time. While our use of a branching process model and the negative binomial distribution provided a first approximation, it failed to capture the heavy-tailed nature and individual-level variability inherent in such networks. Furthermore, the assumption of a static $K$ in our simulations did not reflect the dynamic nature of transmission.

These methodological constraints highlight the need for future studies to incorporate more robust approaches. We recommend combining double-interval censoring methods with multimodal indicators of transmission timing, including symptom onset, precise reporting dates, and viral shedding dynamics. Such hybrid approaches could better account for presymptomatic transmission while minimizing the biases inherent in any single method. Implementation of these approaches will require more comprehensive and standardized data collection, particularly regarding exposure histories and virological characteristics across different transmission contexts.

In conclusion, our study offers important insights into Mpox dynamics, including incubation period, serial interval, pre-symptomatic transmission, and $R_e$. We used various distributions to estimate these parameters, and ongoing surveillance will further enhance our understanding. Our findings emphasize the need for more epidemiological data to refine transmission models and develop more effective control measures. This research provides a solid foundation for public health strategies and highlights the importance of continued research to manage Mpox transmission.

## Supplementary Information

Supplementary Information 1.

## Data Availability

Due to the sensitive nature of the case-level data, original records cannot be shared publicly. However, anonymized datasets containing serial intervals (symptom-onset differences) and incubation periods (exposure-to-onset intervals) are available on GitHub https://github.com/Yehyaalthobaity/Mpox_IP_SI_PT_Rt, alongside all analysis code. The full Mpox dataset is available upon request from the Gulf CDC https://gulfcdc.org/ar/contact-us or the Ministry of Health in the respective Arabian Gulf country where the data originated.
