# Supplementary material for: Exploring the epidemiological characteristics of Mpox in the Arab Gulf countries
Source: Sci Rep. 2025 May 6;15:15748. doi: 10.1038/s41598-025-99252-w (PMC12056212; doi:10.1038/s41598-025-99252-w)
Supplement: Supplementary file 1 — Supplementary Information 1. [file 41598_2025_99252_MOESM1_ESM.pdf]

Exploring the Epidemiological Characteristics of Mpox in the Arab Gulf Countries

Yehya M. Althobaity[1,*], Michael J. Tildesley[2]

1 Department of Mathematics, Taif University, Taif, P.O. Box 11099, Kingdom of Saudi Arabia

2 The Zeeman Institute for Systems Biology and Infectious Disease Epidemiology Research, School of Life Sciences and Mathematics Institute, University of Warwick, Coventry,CV4 7AL, United Kingdom

*Y.mohammed@tu.edu.sa

1 Sensitivity analysis

1.1 Impact of Exposure Window Sensitivity on Incubation Period Estimates

Accurately estimating the incubation period requires defining an appropriate exposure window, which serves as the time interval during which an individual could have been infected. In our main analysis, we assumed a lower bound of 31 days before symptom onset, reflecting a conservative approach to account for potential uncertainties in exposure history. However, the choice of this lower bound can influence the estimated distribution of the incubation period, particularly if a shorter or longer window significantly alters the range of possible exposure times. Given the importance of this assumption, we conducted a sensitivity analysis to assess the robustness of our findings under different exposure window thresholds.

To systematically evaluate the impact of varying the exposure window, we reanalyzed the data using alternative lower bounds, including a 21-day threshold, as suggested by Álvarez et al. [1]. This choice was motivated by prior studies that have used similar or shorter exposure windows, aiming to balance accuracy and data completeness. We applied the same methodological framework as in the main analysis, ensuring consistency in estimation procedures. The results, presented in Table S1, demonstrate that changes in the exposure window led to only slight variations in the estimated incubation period. These findings suggest that our estimates remain stable across reasonable adjustments to the lower bound, reinforcing the robustness of our approach to defining the exposure period.

Table S1: Estimates of the incubation period utilizing gamma, Weibull, and lognormal distributions. Confidence intervals for the shape and scale parameters (logarithmic mean and standard deviation for lognormal) are presented in brackets, representing a 95% confidence level.

**Gamma**	**Mean**	**Shape**	**Rate**	**SD**	**AIC**
	7.93 (6.73 - 9.24)	3.58 (2.30 - 4.52)	2.20 (1.39 - 2.59)	4.16 (2.10 - 5.50)	190.2601
**Weibull**	**Mean**	**Shape**	**Scale**	**SD**	**AIC**
	7.93 (6.85 - 9.31)	1.99 (1.57 - 2.52)	8.95 (7.63 - 10.51)	4.16 (3.95 - 4.46)	191.4717
**Lognormal**	**Mean**	**Mean-log**	**Sd-log**	**SD**	**AIC**
	8.04 (6.73 - 9.60)	1.92 (1.75 - 2.16)	0.54 (0.43 - 0.69)	4.59 (2.84 - 8.59)	191.5112

We assess the incubation period distribution for Mpox using both non-parametric and parametric estimation methods. Specifically, we compare the Kaplan-Meier estimator (nonparametric) with parametric models, including the lognormal, Weibull, and gamma distributions, as shown in Figure S1. This analysis is conducted on stratified data comprising all cases with recorded symptom onset. The figure includes an inset in the top right corner highlighting the density distributions for the gamma, Weibull, and lognormal models.

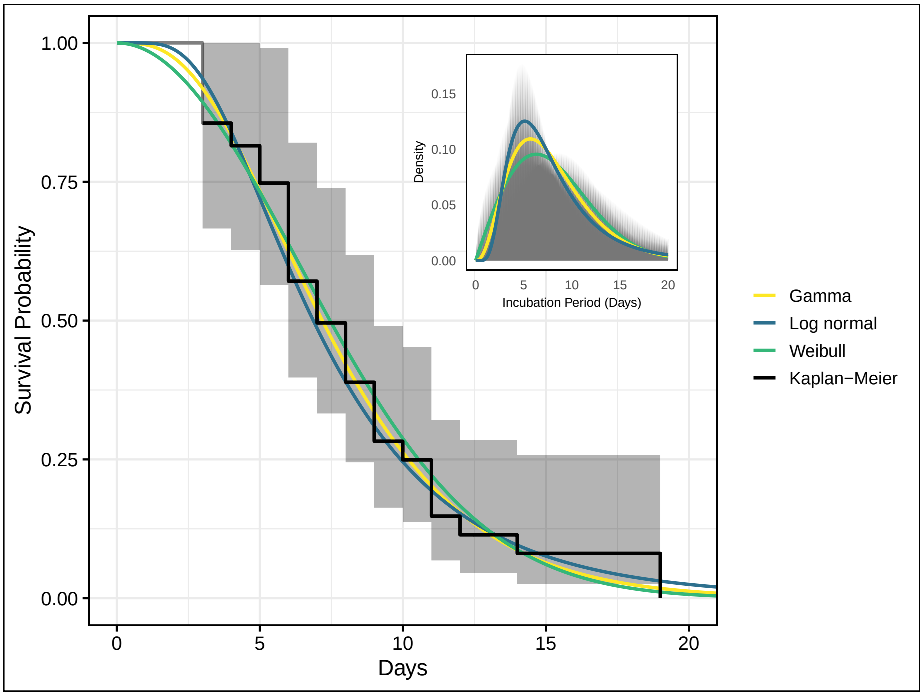

Figure S1: Analyzing the incubation period distribution for Mpox requires comparing nonparametric and parametric estimation methods. The Kaplan-Meier estimator provides a nonparametric survival curve with its 95% confidence interval (CI) (represented by the gray shaded area), while parametric alternatives—including the lognormal, Weibull, and gamma distributions—offer model-based estimates. The inset in the top-right corner displays the corresponding probability density functions for these parametric models, along with their 95% confidence intervals.

1.2 Sensitivity Analysis of R_t Estimates Using the Gamma-Distributed Serial Interval

To evaluate the robustness of our model and better account for uncertainties, we conducted a sensitivity analysis using the same methodological framework as in the main analysis, with one key modification: we assumed a gamma-distributed serial interval. Although [2] recommends the gamma distribution for modeling serial intervals, we derived our parameter estimates directly from our dataset rather than adopting their specific values. This choice was motivated by the gamma distribution's flexibility in capturing the variability observed in serial intervals for Mpox transmission, as supported by previous epidemiologi-

cal studies. In our analysis, the gamma distribution was parameterized with a mean of 7.19 days and a standard deviation of 6.51 days, ensuring consistency with our dataset's characteristics. The shape and rate parameters were estimated based on observed data to accurately reflect the transmission dynamics in our study population. By comparing results across different serial interval assumptions, we further validated the stability and reliability of our findings.

After running the sensitivity analysis with these updated assumptions, we found that the results were very close to those obtained in the main analysis, which strengthens the reliability and robustness of our findings. This consistency across different approaches suggests that the conclusions drawn from the main analysis are not overly sensitive to the choice of serial interval distribution, thereby bolstering confidence in the validity of our estimates.

Specifically, the effective reproduction number R_e, which is crucial for understanding the transmission dynamics of Mpox, was estimated to be 0.97 with a 95% highest posterior density (HPD) interval of 0.91 to 1.28. This estimate indicates that the effective reproduction number remains below the critical threshold of 1, suggesting that the disease is unlikely to spread uncontrollably. These findings are further illustrated in Figure S3. Additionally, we calculated the dispersion parameter K to be 1.55, with a 95% HPD interval ranging from 1.1 to 4.98. This value of K suggests that there is moderate variability in the number of secondary cases per infected individual, providing further insight into the transmission dynamics. The consistency of these parameters across both the main and sensitivity analyses reinforces the robustness and credibility of our model's results.

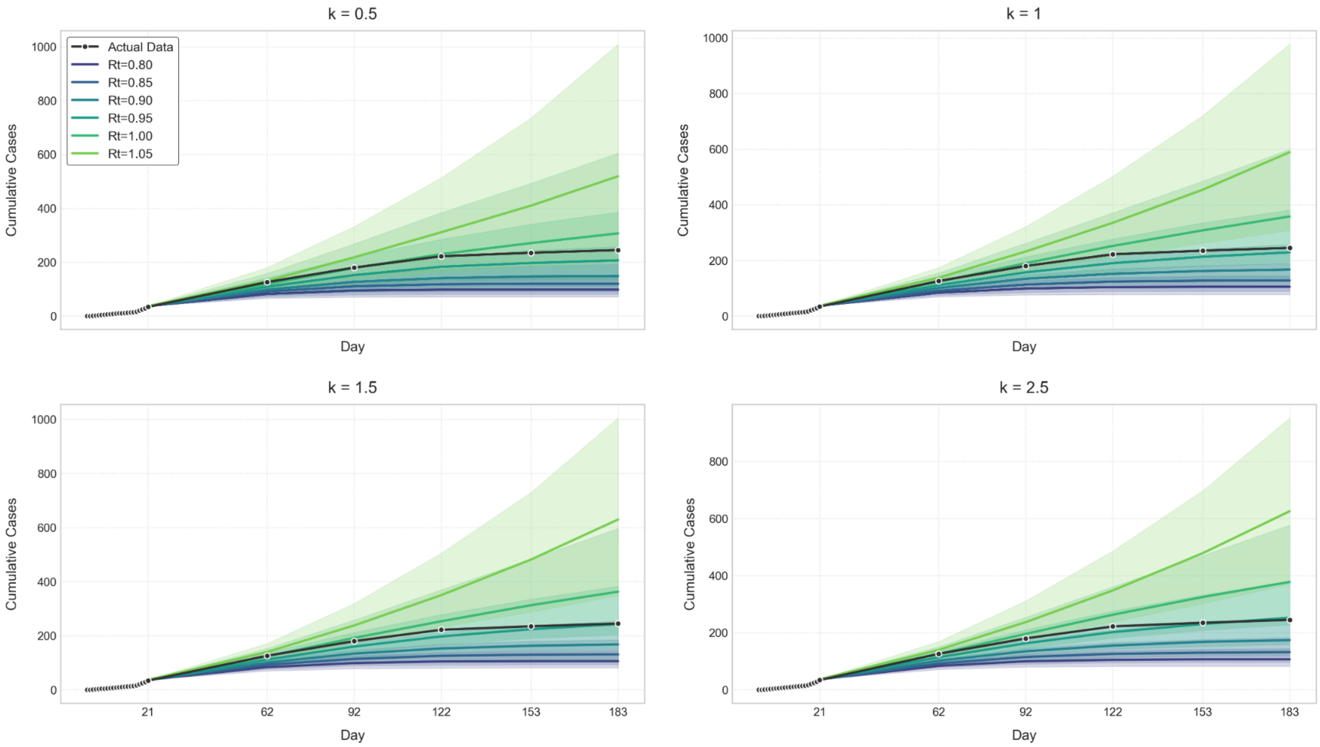

Figure S2: Comparing the cumulative case data with the model projections for the 2022 Mpox epidemic in the AGC provides notable insights. The black lines represent the cumulative case data from June 12, 2022, to January 30, 2023. These data are compared with projections from the individual-instant heterogeneity model, which includes scenarios of homogeneous transmission (panels B-D with $K = 1$, $K = 1.5$, and $K = 2.5$) and heterogeneous transmission (panel A with $K = 0.5$). The colored lines illustrate the median values from the simulated data, reflecting various effective reproduction numbers (R_e). The simulations were conducted using a Weibull-distributed serial interval with a mean of 7.16 days and a standard deviation of 5.89 days, and were iterated 50,000 times.

We employ Markov Chain Monte Carlo (MCMC) sampling to estimate key epidemiological parameters, such as the reproduction number (R_t) and the dispersion parameter (K), using a Bayesian framework. The MCMC sampler used in this function is the No-U-Turn Sampler (NUTS), which is PyMC's default MCMC sampler for continuous models.

NUTS is a variant of Hamiltonian Monte Carlo (HMC) that efficiently explores the parameter space by automatically tuning the step size and path length, eliminating the need for manual adjustments. This is achieved via the `pm.sample` function, which runs three chains with 50,000 samples each. The use of multiple chains helps to assess convergence, with each chain exploring the parameter space independently. Convergence is monitored through trace plots and the effective sample size (see Figure S2).

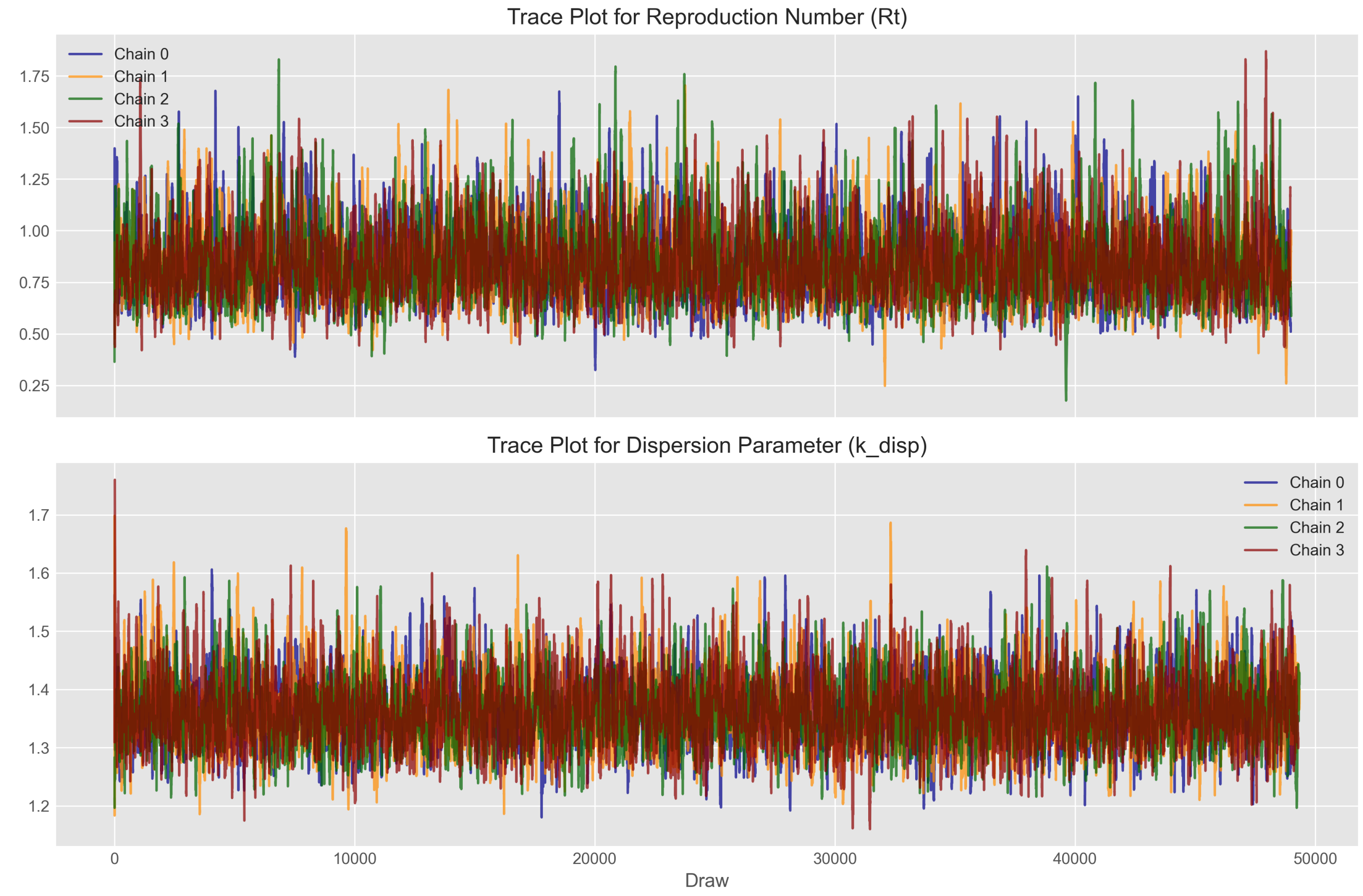

Figure S3: Trace plots for the Markov Chain Monte Carlo (MCMC) sampling of the key epidemiological parameters, R_t and K. The MCMC sampler used is the NUTS (No-U-Turn Sampler), a variant of Hamiltonian Monte Carlo, which efficiently explores the parameter space. Three chains, each with 50,000 samples, were run to assess convergence. The trace plots visualize the parameter chains over iterations, helping to verify the convergence of the sampling process. Convergence is indicated by stable and well-mixed chains across iterations, as observed in these plots, ensuring reliable posterior estimates for the parameters. The effective sample size is also used as an additional diagnostic tool for convergence.

References

[1] Jorge M Estrada Alvarez, Maryluz Hincapié Acuña, Hernán F García Arias, Franklyn E Prieto Alvarado, and Juan J Ospina Ramírez. Estimation of incubation period of mpox during 2022 outbreak in pereira, colombia. *Emerging Infectious Diseases*, 30(1):180, 2024.

[2] Yunjun Zhang and Xiaohua Zhou. Supercritical and homogenous transmission of monkeypox in the capital of china. *Journal of Medical Virology*, 96(2):e29442, 2024.
